# Novel *Silviavirus* phages with broad-host-range activity against methicillin resistant *Staphylococcus aureus* from seafood: comprehending the phenotypic and genotypic variability

**DOI:** 10.1007/s12223-025-01290-4

**Published:** 2025-07-08

**Authors:** Karthika Raveendran, Sifana Sharaf, Ammu Lakshmi D., Reshmi K., Toms Cheriyath Joseph, Raja Swaminathan Thangaraj, Visnuvinayagam Sivam, Shashikanta Parida, Nagendra R. Hegde, Ravindranath Shashidhar, Madhusudana Rao Badireddy, Vandan Nagar, Murugadas Vaiyapuri

**Affiliations:** 1 Microbiology Fermentation and Biotechnology Division, ICAR-Central Institute of Fisheries Technology, Matsyapuri Post, Willingdon Island, Cochin, 682029 Kerala India; 2 Visakhapatnam Research Centre of CIFT, Ocean View Layout, Andhra University P.O, Visakhapatnam, 530003 Andhra Pradesh India; 3 National Institute of Animal Biotechnology, Hyderabad, 500032 Telangana India; 4 Regional Centre for Biotechnology, Faridabad, 121001 USA; 5 Food Technology Division, Bhabha Atomic Research Centre, Trombay, Mumbai, 400085 Maharashtra India; 6 Homi Bhabha National Institute, Mumbai, 400094 Maharashtra India

**Keywords:** *Silviavirus*, Methicillin-resistant *Staphylococcus aureus*, Food safety, Genome analysis, Biocontrol

## Abstract

Methicillin-resistant *Staphylococcus aureus* (MRSA) poses significant challenges to global health, attributed to their ability to resist multiple antibiotic classes. In the current situation, phage-based biocontrol strategies offer a promising alternative, leveraging their high specificity and efficacy against multidrug-resistant bacteria. The present study reports the phenotypic and genotypic characterizations of three broad-host-range MRSA phages: φCIFT_MFB_MRSA12, φCIFT_MFB_MRSA28, and φCIFT_MFB_MRSA32 for their application in seafood safety. The phages exhibited burst sizes ranging from 75 to 107 PFU/cell and burst periods of 80–90 min. The thermal and pH stability studies indicated that φCIFT_MFB_MRSA12 exhibited the highest thermal stability (− 20 to 60 °C), while φCIFT_MFB_MRSA28 demonstrated the widest pH tolerance (pH 3–12). The genomic analysis indicated that the phages possessed linear double-stranded DNA ranging from 141,193 to 141,505 bp, with large direct terminal repeats (DTRs) of 10,893 bp and various coding and non-coding genes (group-I introns, HEARO, and RAGATH). The comparative genome analysis revealed that three phages were found to be closely related to *Silviavirus* phages of the Herelleviridae family and differed with respect to tail fiber proteins, Ig domain-like carbohydrate-binding domains, and certain hypothetical proteins. Interestingly, the intergenomic and phylogenetic analyses revealed that the phages belonged to a novel species. Importantly, the genomes lacked virulence factors, antimicrobial resistance genes, or lysogenic determinants, supporting their safety in biocontrol strategies. The three *Silviavirus* phages could be potential candidates for the biocontrol of MRSA in seafood supply chains, thereby contributing to food safety and security.

## Introduction

Methicillin-resistant *Staphylococcus aureus* (MRSA) is a significant public health concern, causing a plethora of clinical infections and few food-borne illnesses. The infections caused by MRSA accounted for approximately 130,000 deaths in 2021 (Naghavi et al. 2024). Given the declining efficiency of antibiotics, the World Health Organization (WHO) has enlisted *Staphylococcus aureus* as a “high-priority pathogen” emphasizing an urgent action across the one-health sectors to tackle the global menace (Jesudason 2024). The fitness and versatility of MRSA can be attributed to the presence of a wide range of enterotoxins, virulence factors, mobile genetic elements, and an ability to withstand broad temperature ranges (7–48 °C) and high salt stress (10% NaCl) (Ortega et al. 2010; Fisher et al. 2018; Vaiyapuri et al. 2019; Casey and Sleator 2021). MRSA exhibits high genetic diversity, and there is a necessity to have regionally tailored measures to successfully combat the pathogen (Turner et al. 2019). MRSA was once associated with healthcare settings, but now it has spread to livestock, seafood chain, and other agricultural settings largely due to the indiscriminate use of antimicrobial agents (Graveland et al. 2011; Vaiyapuri et al. 2019). MRSA is not an inherent flora of fish and shellfish. Their presence can be attributed to contaminated water, ice, or processing equipment. There are several reports on the incidence of MRSA in fish and fishery products across the world (Visnuvinayagam et al. 2015; Murugadas et al. 2016a; Sivaraman et al. 2022).

Bacteriophages (lytic phages) could serve as potential antimicrobial alternatives due to their abundance in nature, diversity, specificity, broad spectrum activity, low likelihood of resistance development, and ability to disrupt biofilms. In fact, antibiotics have undoubtedly created a revolution in the history of medicine, saving millions of lives, but the fast-nearing menace of bacteria getting ineffective to these antibiotics calls for an urgent need for the development and implementation of alternative strategies against AMR. Phages were already used to treat bacterial infections during the earliest days, but owing to the successful introduction of the antibiotic treatments, phage-based therapeutics lost their importance (Mann 2008; Cui et al. 2024). However, with the growing crisis of antibiotic resistance, phage-based therapeutics are regaining attention as viable alternatives (Rao and Lalitha 2015; Anyaegbunam et al. 2022). Phages are reported to be active in reducing the load of food-borne pathogens and are given generally recognized as safe (GRAS) status for food safety applications (Kahn et al. 2019). The phage-based companies like Intralytix, Inc. (Baltimore, MD, USA), Phagelux (Shanghai, China), and Micreos Food Safety (Wageningen, Netherlands) have been granted FDA approval for their food safety products. Some of the commercial phage preparations against the food pathogens include SalmoPro®, PhageGuard S™, and SalmoFresh™ against *Salmonella* spp.; Ecolicide®, EcoShield™, and Finalyse® against *E. coli* O157:H7; ListShield™ and Phage Guard Listex™ against *Listeria* spp.; and ShigaShield™ against *Shigella* spp. (Moye et al. 2018). Apart from this, some of the phage formulations such as SniPha 360™, Pyophage®, and IntestiPhage® are being used on compassionate grounds in patients. The development highlights the efficacy and safety of phage-based preparations against food-borne pathogens (Endersen and Coffey 2020; Aydin and Can 2020; Gómez-Galindo et al. 2024; Everhart et al. 2025).

The prevalence and epidemiology of MRSA vary geographically, underscoring the need for region-specific collections of phages. In this study, we isolated and comprehensively characterized three lytic phages, φCIFT_MFB_MRSA12, φCIFT_MFB_MRSA28, and φCIFT_MFB_MRSA32, that target MRSA from seafood and aquaculture settings. These phages were analyzed for their host range, growth dynamics, pH and temperature stability, and genotypic features to assess their potential for efficient phage therapy applications.

## Materials and methods

### Sample collection, processing, and storage

Sewage samples (*n* = 10) were collected in sterile glass bottles from various locations in Ernakulam, Kerala, specifically near local fish markets and fish retail shops. Samples were transported under chilled conditions to maintain their integrity. To prepare the samples for analysis, they were centrifuged at 8000 × *g* for 20 min at 4 °C to sediment particulate matter. The supernatant was recovered, stored at 4 °C, and subsequently used for phage screening.

### Bacterial strains and growth conditions

A total of 39 MRSA strains (designated MRSA-1 to MRSA-40) that were earlier isolated from seafood and environment were used in this study whose AMR pattern is detailed in Table 1. By virtue of being identified as MRSA, all the strains tested were resistant to penicillin and cefoxitin. Additionally, they had resistance for either one or two of other classes of antibiotics such as macrolides, quinolones, aminoglycosides, tetracyclines, phenicols, and antimetabolites. These strains were obtained from the repository of ICAR-Central Institute of Fisheries Technology, Kochi, Kerala, and exhibited variations in antimicrobial resistance patterns and sequence types as determined by multilocus sequence typing (MLST) analysis (Visnuvinayagam et al. 2015; Murugadas et al. 2016a, b, 2017a, b, 2020). The bacterial cultures were verified for purity and antibiotic resistance on Mueller–Hinton agar (BD Difco, USA) supplemented with oxacillin (6 µg/mL, Sigma) and NaCl (4%). MRSA strains were also streaked on Baird-Parker agar (BD Difco, USA) enriched with egg yolk potassium tellurite (BD BBL & Difco, USA) to confirm their identity and assess their growth characteristics.

**Table 1:** MRSA isolates used for enrichment of sewage samples to isolate broad-host-range bacteriophages and their resistance profiles

Sl. no	Bacterial strains	Resistance profile
1	MRSA-1	(OX + CX + P) + CIP + E
2	MRSA-2	(OX + CX + P) + CIP + E
3	MRSA-3	(OX + CX + P)
4	MRSA-4	(OX + CX + P)
5	MRSA-5	(OX + CX + P)
6	MRSA-6	(OX + CX + P)
7	MRSA-7	(OX + CX + P) + E
8	MRSA-8	(OX + CX + P) + CIP
9	MRSA-9	(OX + CX + P) + CIP
10	MRSA-10	(OX + CX + P) + CIP + E + COT
11	MRSA-11	(OX + CX + P) + CIP + E + COT
12	MRSA-12	(OX + CX + P) + E
13	MRSA-13	(OX + CX + P) + COT
14	MRSA-15	(OX + CX + P) + E + COT
15	MRSA-16	(OX + CX + P) + COT
16	MRSA-17	(OX + CX + P) + E + COT
17	MRSA-18	(OX + CX + P) + E
18	MRSA-19	(OX + CX + P) + COT
19	MRSA-20	(OX + CX + P) + CIP + GN
20	MRSA-21	(OX + CX + P) + COT
21	MRSA-22	(OX + CX + P)
22	MRSA-23	(OX + CX + P)
23	MRSA-24	(OX + CX + P) + E + GN + TE
24	MRSA-25	(OX + CX + P) + E + C + TE
25	MRSA-26	(OX + CX + P) + E + C + TE
26	MRSA-27	(OX + CX + P) + CIP
27	MRSA-28	(OX + CX + P) + E
28	MRSA-29	(OX + CX + P)
29	MRSA-30	(OX + CX + P) + CIP + E
30	MRSA-31	(OX + CX + P) + CIP
31	MRSA-32	(OX + CX + P)
32	MRSA-33	(OX + CX + P) + CIP + COT
33	MRSA-34	(OX + CX + P) + CIP
34	MRSA-35	(OX + CX + P) + CIP
35	MRSA-36	(OX + CX + P) + E
36	MRSA-37	(OX + CX + P) + CIP + E
37	MRSA-38	(OX + CX + P) + CIP + E
38	MRSA-39	(OX + CX + P) + CIP + E
39	MRSA-40	(OX + CX + P) + CIP + E

Penicillin and its derivates (*P* penicillin, *OX* oxacillin, *CX* cefoxitin), *CIP* ciprofloxacin (quinolones), *E* erythromycin (macrolides), *COT* co-trimoxazole (antimetabolite–folate pathway inhibitors), *GN* gentamycin (aminogylcosides), *TE* tetracycline (tetracyclines), *C* chloramphenicol (phenicols)

### Enrichment of bacteriophages

All 39 MRSA strains were grown in tryptic soy broth (TSB) (BD BBL & Difco, USA) at 37 °C for 16–24 h. A multiple-host enrichment method was employed to enrich bacteriophages from sewage samples (Vaiyapuri et al. 2021). Briefly, 0.5 mL of each overnight-grown MRSA strain was added to flasks containing 5X TSB broth and sewage water samples. The flasks were incubated for 16–24 h at 37 °C for the enrichment of the phages specific to the corresponding bacterial strains. After incubation, the enriched samples were centrifuged at 8000 × *g* for 20 min at 4 °C, and the supernatant was stored at − 20 °C until use (Van Twest and Kropinski 2009; Vaiyapuri et al. 2021).

### Spot assay and purification of phages

Spot assay was performed to detect the presence of phages, by directly spotting 10 µL of each enriched sample filtrate onto the TSA plate spread with 100 µL of the corresponding host bacteria (Carlson 2005). Plates were incubated at 37 °C for 16–24 h and observed for bacterial clearance zones. Lytic spots were picked using a sterile loop and homogenized in saline-magnesium (SM) buffer (50 mM Tris–Cl pH 7.5, 100 mM NaCl, 8 mM MgSO4·7H2O, 0.002% (w/v) gelatin). The sample was centrifuged at 8000 × *g* for 20 min at 4 °C, and the supernatant was serially diluted (10^−1^ to 10^−8^) in SM buffer. Serially diluted samples (1 mL) were mixed with 2 mL of overnight-grown MRSA culture, added to 0.7% soft agar, and plated. Plates were incubated overnight at 37 °C for plaque formation. Selected plaques underwent three to four rounds of purification to isolate morphologically similar plaques. Purified phages were mass-multiplied and stored at − 80 °C.

## Phenotypic characteristics

### Host range analysis

The host range of the phages was assessed against 39 MRSA strains. Overnight-grown cultures were adjusted to 0.5 McFarland turbidity and spread evenly on TSA plates. Ten microliters of each phage suspension was spotted onto plates swabbed with respective bacterial strains (Kutter 2009). The presence of the zones of clearance indicated strong lytic activity. The phage plaque assay was performed using serial dilutions of phages that were spotted on the plates with different bacterial hosts (Kutter 2009; Chhibber et al. 2018).

### Transmission electron microscopy (TEM)

Morphological characterization of the phages was conducted using TEM. Ten microliters of PEG-precipitated phage suspension (10^9^ PFU/mL) was applied to a formvar carbon-coated copper grid (200 mesh), and negatively stained with 2% uranyl acetate (pH 7.4) for 5 min. Grids were washed with sterile nuclease-free water and examined using a JEOL transmission electron microscope (120 kV voltage) at × 15,000 magnification at the National Institute of Animal Biotechnology, Hyderabad, India.

### Growth characteristics

The optimum MOI and adsorption time were determined for each phage, based on which the one-step growth assay was performed (Benala et al. 2021; Kropinski 2009). Briefly, phages were mixed with overnight grown host bacteria at an MOI of 0.1 and incubated for 10 min at 37 °C for adsorption. The one-step growth curve was determined with slight modifications to the protocol described by Ellis and Delbruck (1939). This mixture was diluted fourfold, and aliquots were plated on soft agar at 10-min intervals up to 90 min. Plates were incubated at 37 °C for 16–24 h, and phage titers were determined. A growth curve was generated by plotting the phage titer on the *Y*-axis and time on the *X*-axis. The latent period, burst size, and burst period of the phages were determined using the growth curve. The burst size was estimated by dividing the phage titer at the end of the burst phase by the concentration of host bacteria at the beginning of infection (Gao et al. 2024).

### pH stability

The pH stability studies were carried out with slight modifications to the protocol described previously (Gao et al. 2024). The effect of pH on the titer and viability of the phages was evaluated by incubating 100 µL of the phage suspension in 900 µL of SM buffer adjusted to different pH levels (pH 3–12). The changes in titer of the phages were determined by comparing to a control (pH 7).

### Thermal stability studies

The thermal stability of the phages was assessed by incubating the phage suspension at varied temperatures ranging from − 20 to 60 °C for 2 h (Soro et al. 2024). Phage titers were compared to those stored at − 20 °C to evaluate their thermal stability.

### Genome sequencing, assembly, and annotation

Phage samples were scaled up and precipitated using polyethylene glycol (PEG) 8000/1 M NaCl (Yamamoto et al. 1970). DNA was extracted using the DNeasy Blood and Tissue Kit (Qiagen, USA), following DNase I and RNase A treatment and proteinase K digestion as described by Jakočiūne and Moodley (2018). DNA quality and concentration were assessed using a nano-spectrophotometer, and integrity was verified on 1.2% agarose gel. Whole-genome shotgun sequencing of φCIFT_MFB_MRSA12, φCIFT_MFB_MRSA28, and φCIFT_MFB_MRSA32 was performed on the Illumina NovaSeq 6000 platform at Lifecell International Pvt. Ltd., Chennai, India. Raw data quality was assessed using FastQC v0.11.9 and MultiQC v1.9 (Andrews 2010; Ewels et al. 2016). Adapter sequences and low-quality bases were trimmed using fastp v0.12.4 (Chen et al. 2018), and taxonomic classification was conducted using Kraken2 v2.1.2 (Wood and Salzberg 2014). Pre-processed reads were assembled de novo on the Galaxy platform using Shovill (Galaxy v1.1.0 + galaxy2) (Giardine et al. 2005; Blankenberg et al. 2010). Genome statistics were evaluated with QUAST v5.0.2 (Gurevich et al. 2013), and BLAST v2.13.0 was used for sequence comparison against the NCBI nt database (Altschul et al. 1990). Annotation was performed with Prokka v1.13 (Seemann 2014) and phage-specific gene prediction was made using PHANOTATE (McNair et al. 2019).

Phagescope, incorporating the tRNAscan-SE v2.0, ARAGORN v1.2.41, AcRanker, CRISPRCasFinder v4.2.20, and CARD, was used for the prediction of phage lifestyle, tRNA, tmRNA genes, anti-CRISPR proteins, CRISPR arrays, virulent factors, and antimicrobial resistance genes (Laslett and Canback 2004; McArthur et al. 2013; Eitzinger et al. 2020; Chan et al. 2021; Wang et al. 2024). ABRicate and Integron Finder provided in the galaxy pipeline (Galaxy Version 2.0.5 + galaxy0) were further screened for the presence of virulence, AMR, and lysogeny-related genes (Seemann 2016; Néron et al. 2022). The viral phylogenetic tree (ViPTree) was used to generate a proteomic tree of phages, identifying the closest taxa (Nishimura et al. 2017). Virus Intergenomic Distance Calculator (VIRIDIC) and taxmyPHAGE that follow International Committee on Taxonomy of Viruses (ICTV) guidelines were used to calculate and visualize the virus intergenomic similarities (Moraru et al. 2020; Millard et al. 2024). Virus Classification and Tree Building Online Resource (VICTOR) was used to infer the phylogenetic relationships of the three phages to other *Silviavirus* phages and their classification (Meier-Kolthoff and Göker 2017). Genomic rearrangements were determined using progressiveMauve (Darling et al. 2004), the complete genome sequence similarities were visualized using BLAST Ring Image Generator (BRIG) (Alikhan et al. 2011), and the phage termini/packaging mechanisms were interpreted by PhageTerm (Garneau et al. 2017).

## Results

### Morphological and biological characterization of the phages

**Fig. 1:**
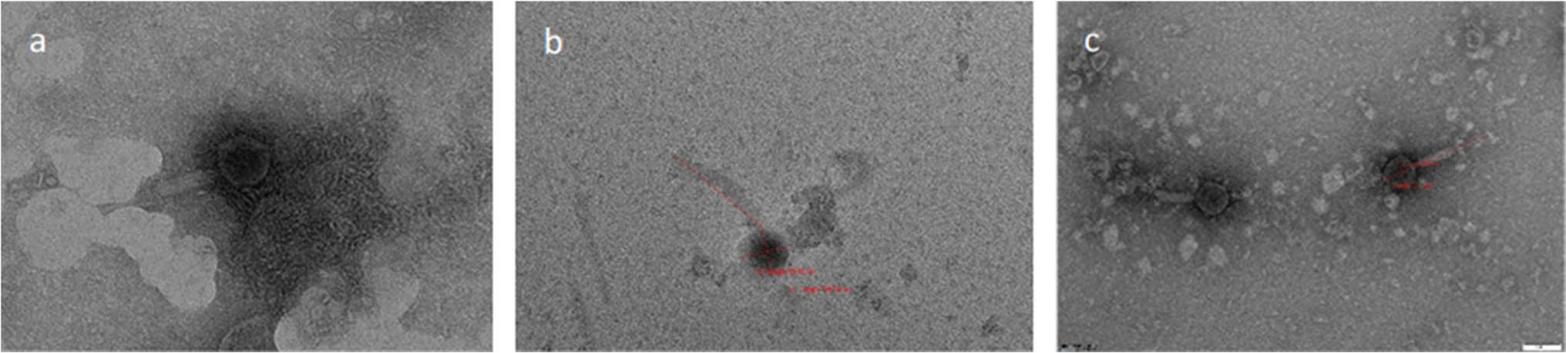
Transmission electron micrographs (TEM) of phages, **a** φCIFT_MFB_MRSA32, **b** φCIFT_MFB_MRSA28, and **c** φCIFT_MFB_MRSA12 negatively stained with 2% (w/v) uranyl acetate (scale 100 nm)

Three lytic phages, viz., φCIFT_MFB_MRSA12, φCIFT_MFB_MRSA28, and φCIFT_MFB_MRSA32, were isolated and purified using MRSA-12, MRSA-28, and MRSA32 as hosts, respectively. The phages formed clear plaques with a diameter of 1–2 mm. The phages were named after the original hosts used for their isolation and purification. The transmission electron microscopic (TEM) analysis indicated distinct morphological characters. Phage φCIFT_MFB_MRSA12 had a head measuring ~ 90 ± 5.4 nm and a tail measuring ~ 216 ± 2.31 nm long, φCIFT_MFB_MRSA28 had a head ~ 89 ± 0.57 nm in diameter and a ~ 210 ± 2.72 nm tail, and φCIFT_MFB_MRSA32 displayed a head ~ 80 nm ± 1.12 nm in diameter with a ~ 289 ± 4.94 nm tail. All the three phages exhibited myoviral morphology, characterized by an icosahedral head and a long contractile tail (Fig. 1).

**Fig. 2:**
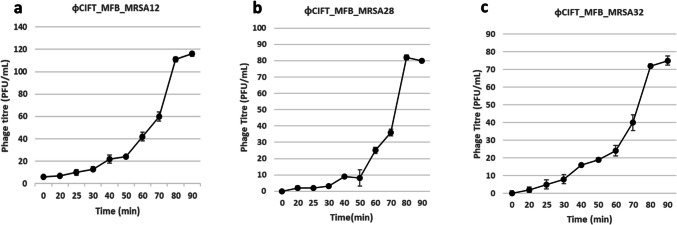
One step growth curve of the phages **a** φCIFT_MFB_MRSA12, **b** φCIFT_MFB_MRSA28, and **c** φCIFT_MFB_MRSA32. Time 0 represents the phage titer measured immediately after a 10-min adsorption period. The *Y*-axis shows the normalized phage titer (PFU/mL), while the *X*-axis indicates the time intervals for the study

The host range of the three phages was assessed initially using the spot test, where their ability to form lytic zones on lawns of 39 MRSA strains was evaluated. Further, the lytic spots were purified, serially diluted, and spotted on a range of bacterial hosts. The results showed that φCIFT_MFB_MRSA12, φCIFT_MFB_MRSA28, and φCIFT_MFB_MRSA32 were active against 76.9% (30/39), 82.1% (32/39), and 71.8% (28/39) of the host strains, respectively. The growth parameters of the phages were analyzed using a single-step growth curve at a MOI of 0.1, which was standardized using a microtiter based method in a 4-h period. The adsorption assay performed indicated that 80–90% of the phages adsorbed to their hosts within the first 10 min. The latency period, defined as the time between adsorption and the first burst, was approximately 30–50 min, while the burst period lasted 70–90 min. The burst sizes were around 107 PFU/cell for φCIFT_MFB_MRSA12, 82 PFU/cell for φCIFT_MFB_MRSA28, and 75 PFU/cell for φCIFT_MFB_MRSA32 (Fig. 2). The host range of the three phages was assessed using a spot test, where their ability to form lytic zones on lawns of 39 MRSA strains was evaluated. The results showed that φCIFT_MFB_MRSA12, φCIFT_MFB_MRSA28, and φCIFT_MFB_MRSA32 were active against 76.9% (30/39), 82.1% (32/39), and 71.8% (28/39) of the host strains, respectively (Table 2).

**Table 2:** The phenotypic characteristics of the three phages, including the host range, morphology, and burst size, are indicated below

Phage	Host range	Morphology	Burst size (PFU/cell)
φCIFT_MFB_MRSA12	76.9%	Icosahedral head (~ 90 ± 5.4 nm) Long contractile tail (~ 216 ± 2.31 nm)	107
φCIFT_MFB_MRSA28	82.1%	Icosahedral head (~ 89 ± 0.57 nm) Long contractile tail (~ 210 ± 2.72 nm)	82
φCIFT_MFB_MRSA32	71.8%	Icosahedral head (~ 80 ± 1.12 nm) Long contractile tail (~ 289 ± 4.94 nm)	75

**Fig. 3:**
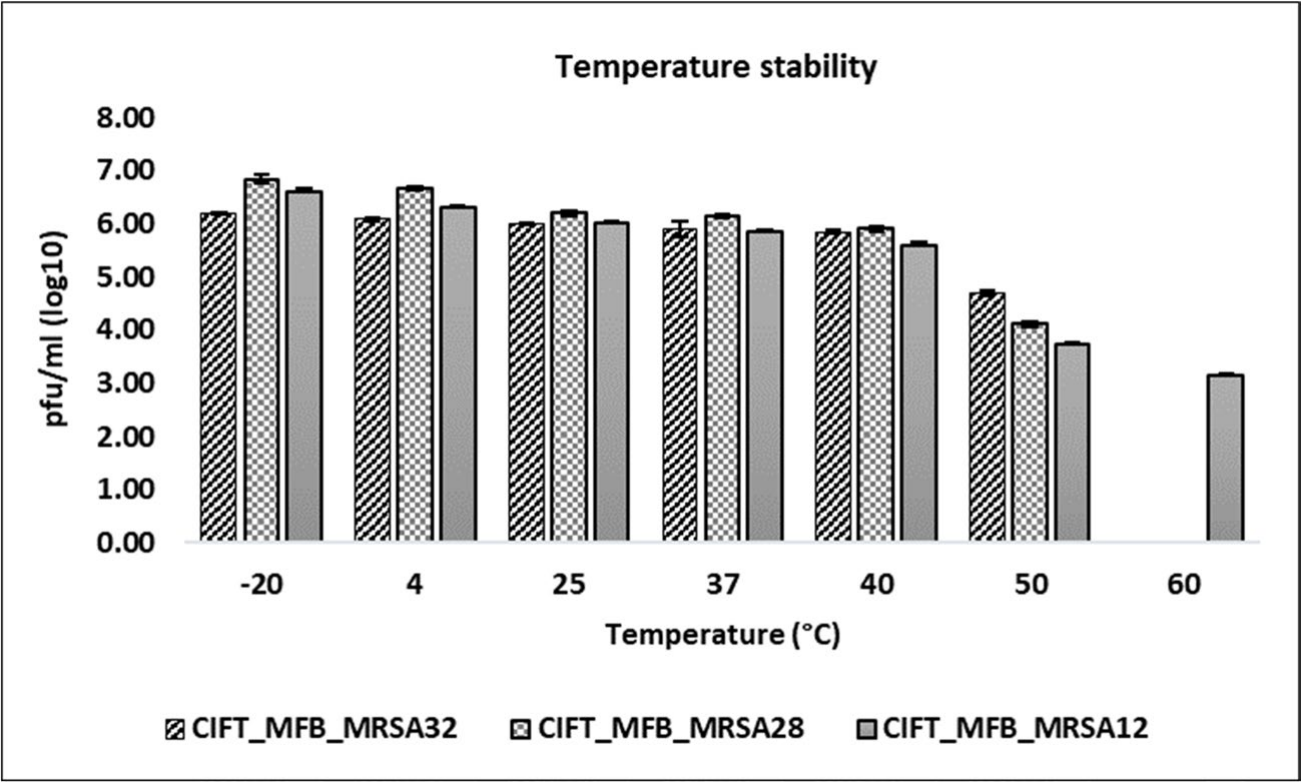
The thermal stability of phages φCIFT_MFB_MRSA12, φCIFT_MFB_MRSA28, and φCIFT_MFB_MRSA32 assessed across a temperature range of − 20 to 60 °C. The titer of the phages is indicated as the fold change compared to control and is presented as means ± SD. At higher temperatures, the phage titers showed a considerable decline in the titer (*p* < 0.05)

Thermal stability of the phages was evaluated over a temperature range of − 20 to 60 °C for 2 h. The results showed that the phage titers gradually decreased with increasing temperature, with the highest stability observed at − 20 °C (*p* < 0.05). Phage φCIFT_MFB_MRSA12 remained viable at 60 °C, though a 3.5 log reduction in PFU/mL was noted compared to the − 20 °C samples. The phages φCIFT_MFB_MRSA28 and φCIFT_MFB_MRSA32 maintained stability between − 20 and 50 °C, but their viability was completely lost at 60 °C (Fig. 3).

**Fig. 4:**
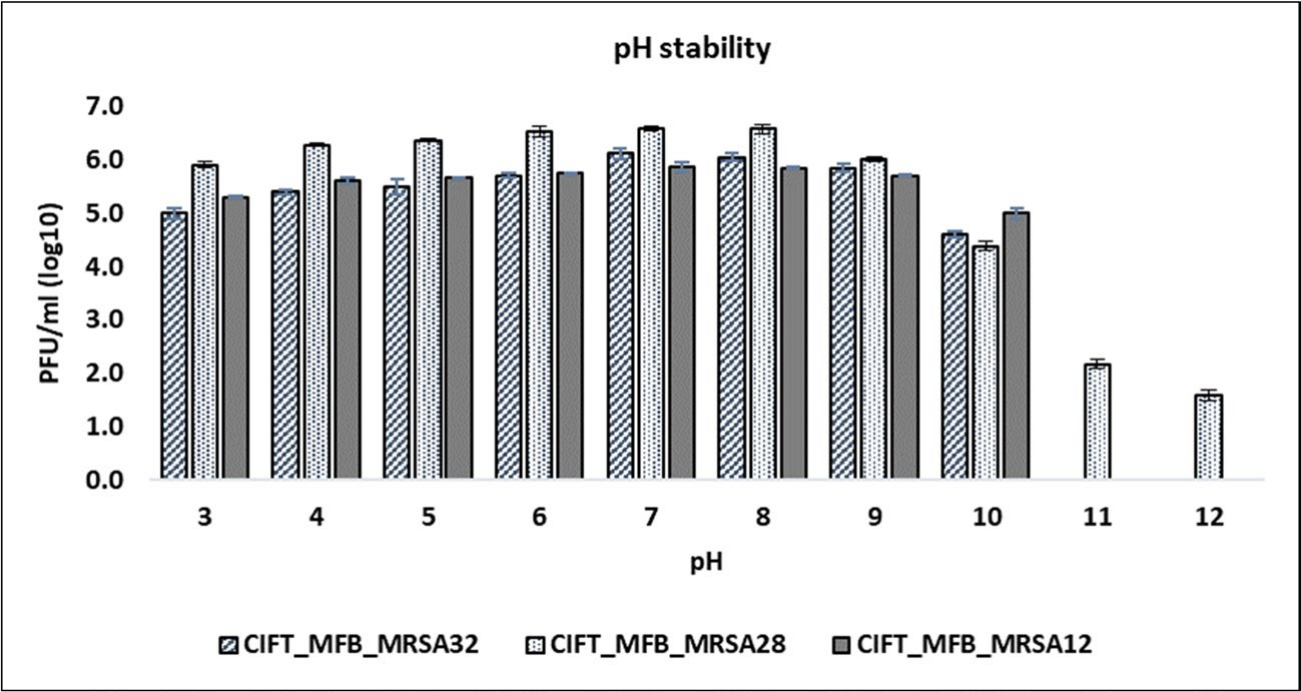
The stability of phages φCIFT_MFB_MRSA12, φCIFT_MFB_MRSA28, and φCIFT_MFB_MRSA32 across the pH range of 3 to 12. The titer of the phages is indicated as the fold change compared to control and is presented as means ± SD. At higher pH range, the phage titers showed a considerable decline (*p* < 0.05)

The pH tolerance test showed that phage φCIFT_MFB_MRSA28 remained viable across the entire tested pH range (3–12), though a 5 log PFU/mL reduction was observed at pH 12 and a 0.7 log PFU/mL reduction at pH 3. The titer gradually decreased as the pH deviated from the optimal range. In contrast, phages φCIFT_MFB_MRSA32 and φCIFT_MFB_MRSA12 were more sensitive to pH increases, with no visible plaques at pH 11–12 (*p* < 0.05) (Fig. 4).

### Genome features and annotation

The genome sequencing data indicated that more than 90% of the reads had a quality score greater than Q30. Whole-genome shotgun sequencing of phages φCIFT_MFB_MRSA12, φCIFT_MFB_MRSA28, and φCIFT_MFB_MRSA32 revealed genomes of similar sizes: 141,193 bp, 141,344 bp, and 141,505 bp, respectively. All three phages contained linear dsDNA genomes with long direct terminal repeats (DTRs) of 10,893 bp. Phages φCIFT_MFB_MRSA12 and φCIFT_MFB_MRSA32 each had 193 ORFs, while φCIFT_MFB_MRSA28 contained 194 ORFs. Genome details are summarized in Table 3. BLASTn pairwise sequence alignment showed that the phages were 99.9% identical, classifying them as isolates of the same species (Adriaenssens and Brister 2017). The top BLASTn hits for all three phages were related to the *Silviavirus* genus.

**Table 3:** Genome details of phages φCIFT_MFB_MRSA12, φCIFT_MFB_MRSA28, and φCIFT_MFB_MRSA32

Genome details of phages	φCIFT_MFB_MRSA12	φCIFT_MFB_MRSA28	φCIFT_MFB_MRSA32
GenBank accession number	PQ311674	PV017447	PV017448
SRA accession number	SRR30027825	SRR30200128	SRR30200129
Genome size	141,193 bp	141,344 bp	141,505 bp
Genome	Linear dsDNA	Linear dsDNA	Linear dsDNA
DTRs	10,893 bp	10,893 bp	10,893 bp
ORFs	193	194	193
CDS	185	186	185
G + C	29.93%	29.93%	29.91%
tRNA/tmRNA	NIL	NIL	NIL
Lifestyle prediction	Virulent	Virulent	Virulent
Virulence genes	NIL	NIL	NIL
Antimicrobial resistance genes	NIL	NIL	NIL
ncRNAs	8	8	8

The annotated ORFs were classified into the following categories depending on their functions: (1) DNA replication and repair—DNA binding proteins, recombinase, DNA polymerase, resolvase, DNA primases, and DnaB-like replicative helicase; (2) transcription—antirepressor, zinc finger domain protein, RNA polymerase sigma factor, and antisigma factor; (3) nucleotide metabolism—nucleoside deoxyribosyltransferase, DNA methyl transferase, RinB-like transcriptional activator, ribonucleotide reductase, and glycerophosphoryl diester phosphodiesterase; (4) DNA packaging—terminase large subunit, terminase small subunit, HNH endonuclease, and portal protein; (5) structural proteins—virion structural protein, major capsid protein, minor capsid protein, major tail sheath protein, base plate protein, tail fiber protein, tail assembly chaperone, tail sheath proteins, head maturation protease, and major tail protein; (6) host cell lysis—endolysin, holin, tail associated lysin, tail protein with lysin activity, and N-acetylmuramoyl-L-alanine amidase; (7) hypothetical proteins; (8) noncoding RNAs; and (9) others—transposases, thioredoxin-like protein, and pentapeptide repeat family protein. The noncoding RNAs identified in the phage genomes included group I introns, HEARO (HNH endonuclease-associated RNA and ORF), and RAGATH-18 (RNAs associated with genes associated with twister and hammerhead ribozymes) (Table 4). Remarkably, no genes encoding tRNA, bacterial virulence factors, or antimicrobial resistance-related functions were identified.

**Table 4:** The various non-coding RNA identified in the genomes of the three phages

Phage	Group I introns	CDS with intron overlap	HEARO	RAGATH
φCIFT_MFB_MRSA12	12_ORF151	12_ORF152	12_ORF133	12_ORF166
	12_ORF178	12_ORF182		12_ORF19
	12_ORF179			
	12_ORF180			
	12_ORF181			
φCIFT_MFB_MRSA28	28_ORF16	28_ORF15	28_ORF34	28_ORF169
	28_ORF183	28_ORF182		28_ORF1
	28_ORF184			
	28_ORF185			
	28_ORF186			
φCIFT_MFB_MRSA32	32_ORF128	32_ORF129	32_ORF111	32_ORF172
	32_ORF155	32_ORF159		32_ORF143
	32_ORF156			
	32_ORF157			
	32_ORF158			

Although the phages exhibited similar genome sizes, differences in host range were observed. To explore these differences, the ORFs of the three phages were compared. It was found that certain ORFs present in one phage genome were absent in others. The phage φCIFT_MFB_MRSA28, which displayed a comparatively larger host range, contained unique ORFs such as 28_ORF172 (tail fiber protein: 750 bp), 28_ORF26 (DNA methyltransferase: 177 bp), and 28_ORF194 (hypothetical protein: 96 bp). Additionally, ORFs present in φCIFT_MFB_MRSA12 and φCIFT_MFB_MRSA32 but absent in φCIFT_MFB_MRSA28 were identified. Differences were also noted between φCIFT_MFB_MRSA12 and φCIFT_MFB_MRSA32, with unique ORFs in each (Table 5a–d).

**Table 5:** The three phages were compared to each other, and the ORFs that showed differences between the phages are indicated: (a) ORFs absent in φCIFT_MFB_MRSA28 but present in φCIFT_MFB_MRSA12, (b) ORFs absent in φCIFT_MFB_MRSA28 but present in φCIFT_MFB_MRSA32, (c) ORFs absent in φCIFT_MFB_MRSA32 but present in φCIFT_MFB_MRSA12, and (d) ORFs absent in φCIFT_MFB_MRSA12 but present in φCIFT_MFB_MRSA32

	ORFs	Protein product	Length (bp)
(a)	12_ORF1	Hypothetical protein	234
	12_ORF107	Ig domain containing protein	633
	12_ORF162	IS200/IS605 family transposase ISBth15	1221
(b)	32_ORF91	Ig domain containing protein	633
	32_ORF178	Membrane protein	696
	32_ORF146	IS200/IS605 family transposase ISBth15	1221
(c)	12_ORF1	Hypothetical protein	234
(d)	32_ORF178	Membrane protein	696

### Comparative genomics and phylogenetic studies

**Fig. 5:**
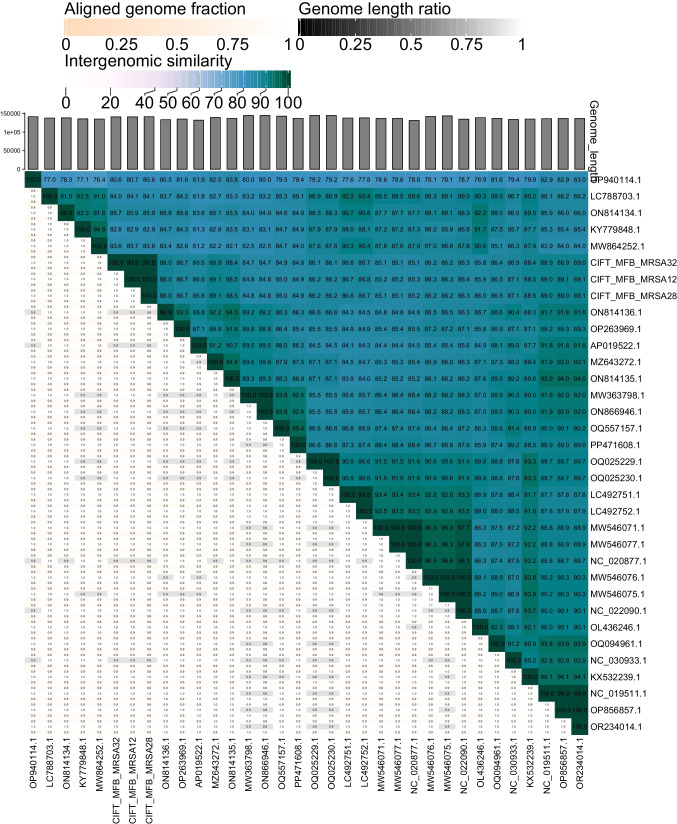
Pairwise intergenomic similarities of the three phages calculated using VIRIDIC with other closely related members of *Silviavirus* genus. The percentage of homology is represented by the numbers in the chart

**Fig. 6:**
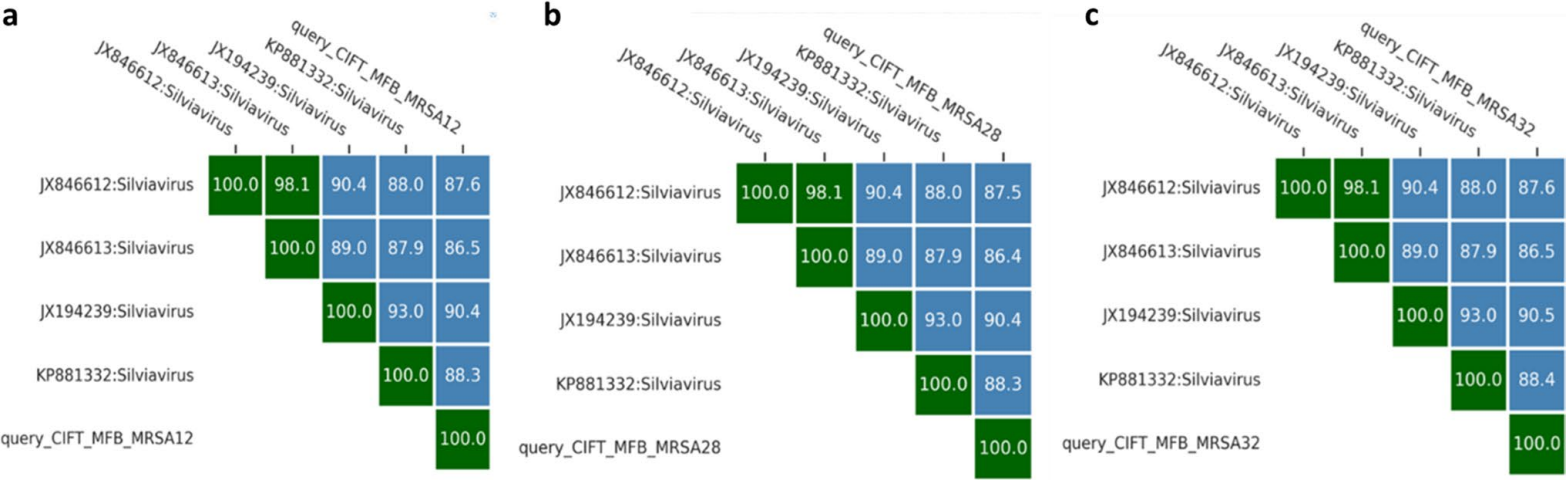
The taxMyPHAGE-based prediction of pairwise intergenomic similarities of the three phages **a** φCIFT_MFB_MRSA12, **b** φCIFT_MFB_MRSA28, and **c** φCIFT_MFB_MRSA32 with closely related *Silviavirus* phages

**Fig. 7:**
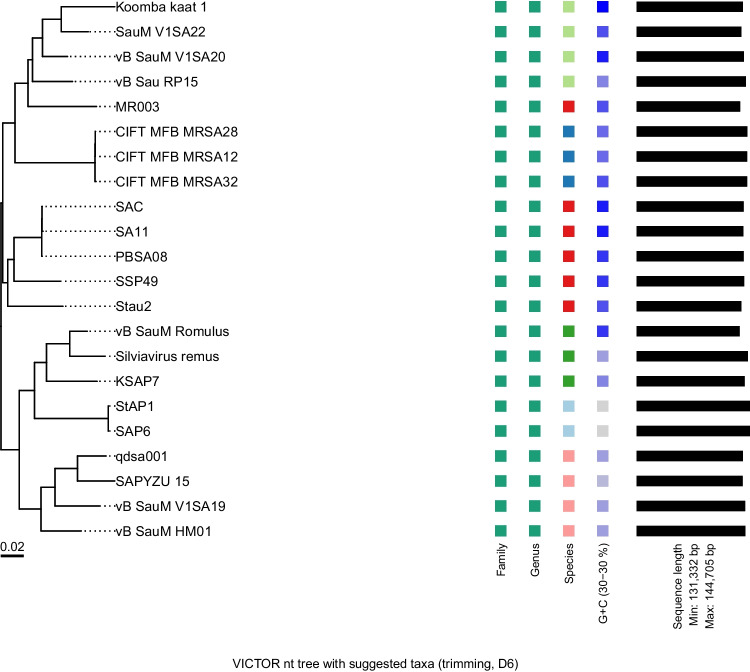
The Genome BLAST Distance phylogenetic tree constructed using 22 whole-genome sequences by VICTOR, with default settings. The study shows that the phages φCIFT_MFB_MRSA12, φCIFT_MFB_MRSA28, and φCIFT_MFB_MRSA32 are novel species in the *Silviavirus* genus

Comparative genomic analysis revealed that the three phages φCIFT_MFB_MRSA12, φCIFT_MFB_MRSA28, and φCIFT_MFB_MRSA32 shared approximately 99.9% homology based on their average nucleotide identity (ANI), as calculated using JSpeciesWS (Richter et al. 2016). Despite this high similarity, differences were observed in certain coding sequences (CDSs), including those encoding tail fiber proteins, hypothetical proteins, and immunoglobulin (Ig) domain-like carbohydrate-binding domains. BLASTn analysis indicated 93–98% nucleotide identity over 83–91% query coverage compared to *Staphylococcus* phages belonging to the *Silviavirus* genus, subfamily Twortvirinae, family Herelleviridae, and class Caudoviricetes. Pairwise intergenomic similarities calculated using VIRIDIC, comparing the phages to closely related *Silviavirus* members, demonstrated the highest similarity (89%) to *Staphylococcus phage* SAC, *Staphylococcus* phage PBSA08, and *Staphylococcus* phage vB_Sau_RP15. The lowest similarity (80%) was observed with *Staphylococcus* phage vB_SauM_VL10 (Fig. 5). The VIRIDIC analysis indicated that the phages shared less than 95% similarity with other *Silviavirus* species, suggesting their classification as a distinct species within the genus. These findings were corroborated by taxmyPHAGE analysis, which also suggested that the phages might constitute a new species (Fig. 6). Additionally, a Genome-BLAST Distance Phylogeny (GBDP) tree generated using VICTOR further supported this conclusion, clustering φCIFT_MFB_MRSA12, φCIFT_MFB_MRSA28, and φCIFT_MFB_MRSA32 as a separate species within the *Silviavirus* genus (Fig. 7). Consistent with the criteria established by the ICTV for novel species identification, the similarity of the three phages to other *Silviavirus* phages fell below the species threshold of 5%. Thus, φCIFT_MFB_MRSA12, φCIFT_MFB_MRSA28, and φCIFT_MFB_MRSA32 have been classified as a novel species within the S*ilviavirus g*enus.

**Fig. 8:**
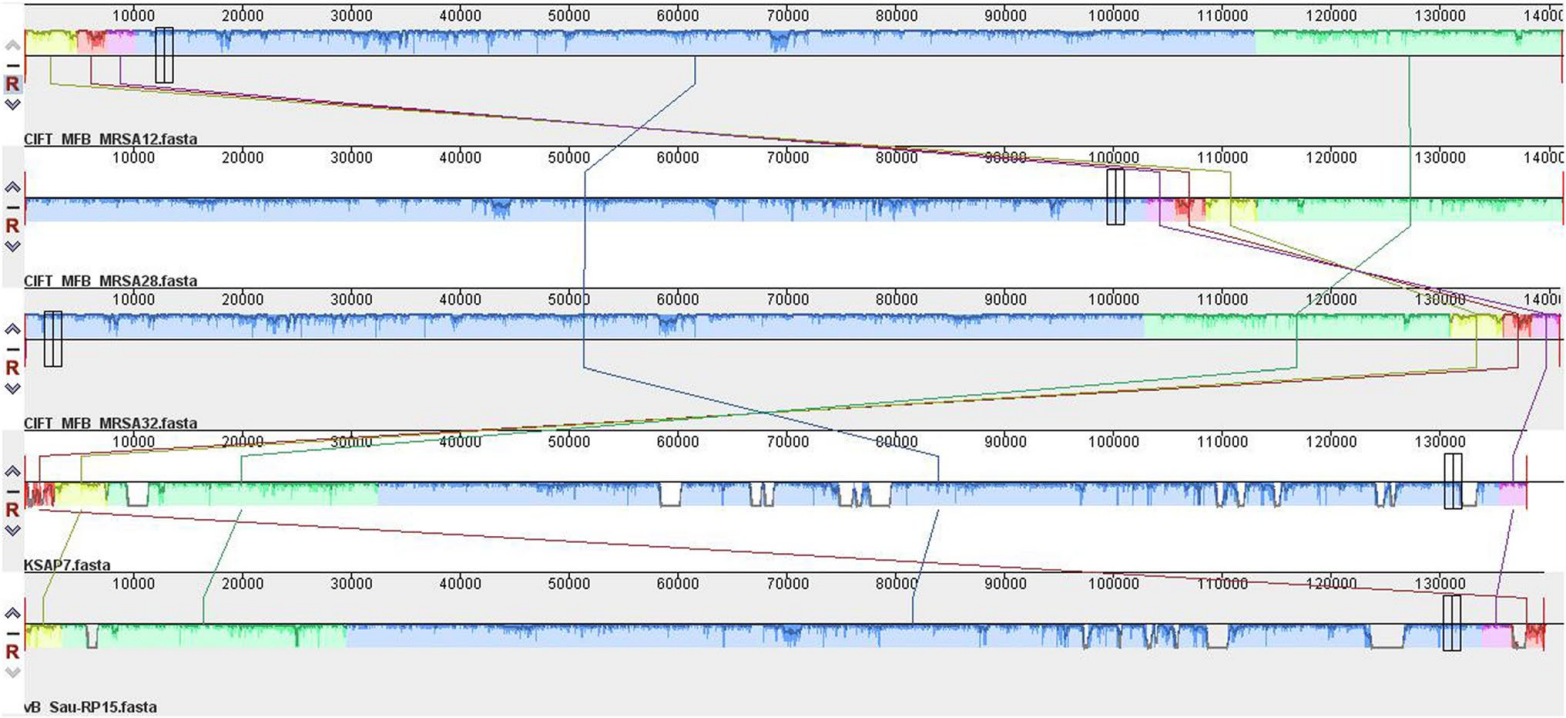
Progressive Mauve analysis of the phages φCIFT_MFB_MRSA12, φCIFT_MFB_MRSA28, and φCIFT_MFB_MRSA32 along with two *Silviavirus* genomes, viz., *Staphylococcus* phage KSAP7 and *Staphylococcus* phage vB_Sau-RP15 using default settings. There are five locally collinear blocks (LCBs) which have undergone gene rearrangements. The LCBs of the phage φCIFT_MFB_MRSA28 are in reverse complement orientation, similar to *Staphylococcus* phage KSAP7 and *Staphylococcus* phage vB_Sau-RP15, whereas φCIFT_MFB_MRSA32 and φCIFT_MFB_MRSA12 were arranged in forward orientation

**Fig. 9:**
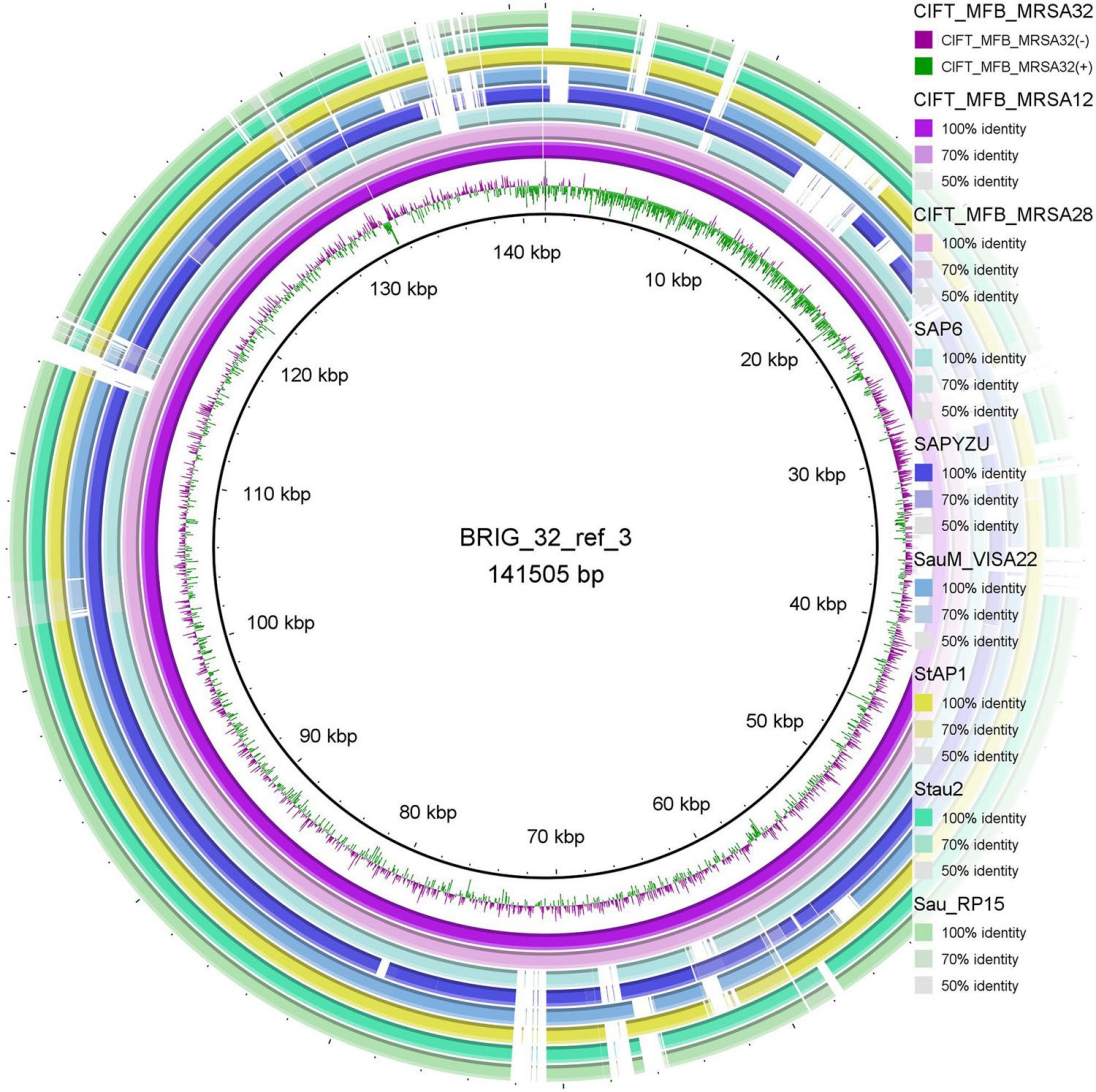
BLAST Ring Image Generator (BRIG, version 0.95) depicting the circular genome comparisons among φCIFT_MFB_MRSA12, φCIFT_MFB_MRSA28, and φCIFT_MFB_MRSA32, and other closely related *Silviavirus* phage genomes

**Fig. 10:**
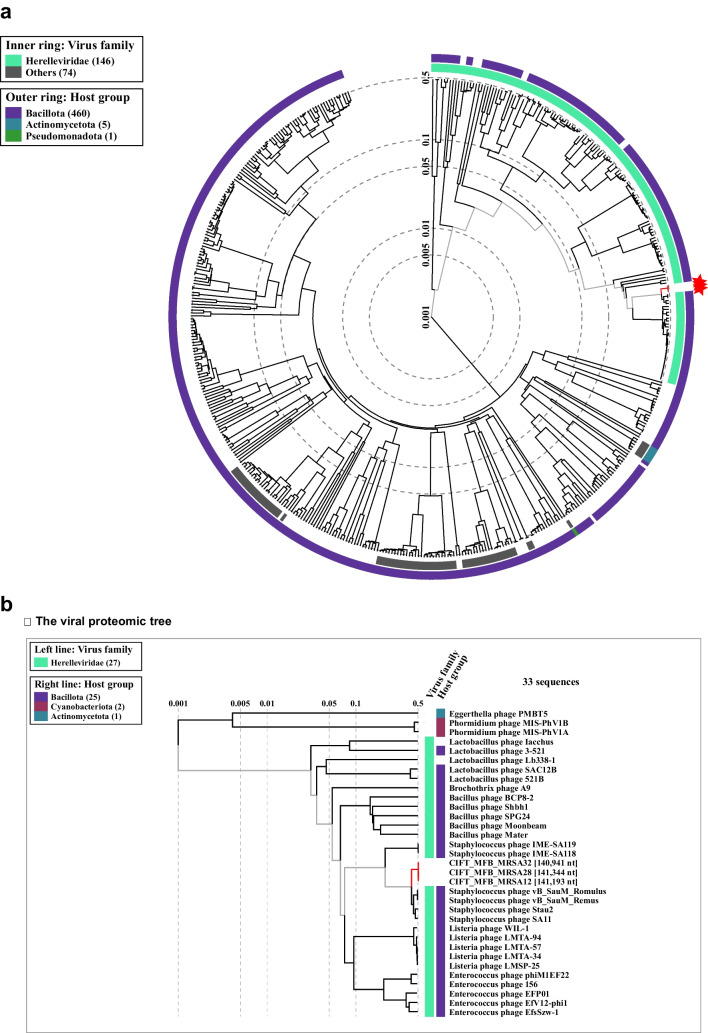
Viral phylogenetic tree (ViPTree) analysis based on genome-wide sequence similarities. **a** ViPTree of φCIFT_MFB_MRSA12, φCIFT_MFB_MRSA28, and φCIFT_MFB_MRSA32 and other phage genomes in the ViPTree database presented in the circular view, where the colored rings represent the virus families (inner ring) and host groups (outer ring). The three studied phage genomes are marked with a red asterisk. **b** The rectangular phylogenetic tree (subset) of the phages generated using ViPTree, with the log scale on top. Red branches represent the three phage genomes studied, while black branches represent linear dsDNA known phage genomes from the ViPTree database. The classification of the phages based on the host group and family level is represented by the colored right and left lines

MAUVE analysis was performed to predict the genome arrangements of the three highly similar phages and compared them to two phage genomes downloaded from NCBI, namely *Staphylococcus* phage KSAP7 and *Staphylococcus* phage vB_Sau-RP15, using default settings (Fig. 8). Five collinear blocks were identified across the genomes. BRIG analysis was conducted to visualize circular genome-level comparisons between the three phages and other NCBI-listed phages (Fig. 9). ViPTree analysis further demonstrated that the three phages clustered closely with *Silviavirus* phages while branching distinctly from Kayvirus phages. Additionally, the three phages were grouped with other members of the Herelleviridae family (Fig. 10).

## Discussion

The advancements in modern medicine face an imminent threat from the emergence of antimicrobial resistance (AMR), a critical global public health issue of the twenty-first century. Antimicrobial resistance was responsible for an estimated 1.27 million attributed deaths in 2019 (Murray et al. 2022; Naghavi et al. 2024). Methicillin-resistant *Staphylococcus aureus* (MRSA) ranks as the second leading pathogen for antibiotic resistance-associated deaths and is listed by the WHO in its Bacterial Priority Pathogens List (BPPL) as requiring urgent alternatives to antibiotics (Jesudason 2024; Murray et al. 2022). There is an imperative need for new antimicrobials that are effective in limiting the growth of these harmful bacteria and promoting sustainable healthcare development. Lytic phages could serve as a promising tool to combat the menace of AMR, as they are more specific and self-limiting and have a reduced chance of resistance development as they can coevolve with their hosts (Koskella and Brockhurst 2014; Piel et al. 2022). In the present study, three lytic Caudovirales phages, viz., φCIFT_MFB_MRSA12, φCIFT_MFB_MRSA28, and φCIFT_MFB_MRSA32, were isolated from sewage samples obtained from the markets of Kochi, Kerala. These phages were found to target the MRSA strains isolated from seafood markets and aquaculture settings. The isolated phages displayed a broad host range (71–82%), which makes them desirable candidates for phage therapy research.

The effect of varied environmental conditions on the stability of the phages was assessed for their suitability for therapeutic applications. The thermal stability studies conducted at a temperature range between − 20 and 60 °C indicated that there was a gradual reduction in phage titers with an increase in temperature, with the highest stability exhibited at − 20 °C. Of the three phages, only φCIFT_MFB_MRSA12 retained viability at 60 °C. The pH stability studies conducted within a pH range of 3–12 indicated that the optimum viability was found to be at the pH range of 7–8. While the phage φCIFT_MFB_MRSA28 was viable across all the pH ranges (3–12) tested, the phages φCIFT_MFB_MRSA32 and φCIFT_MFB_MRSA12 lost their viability at pH 11–12. The one-step growth curve analysis demonstrated that the latent period of the phages was around 30–50 min, with an average burst size of ~ 75–107 plaque-forming units (PFU) per infected cell. Phages belonging to the Herelleviridae family were previously reported to exhibit an average latent period ranging from 30 to 50 min, and the findings from the present study align with those of Lerdsittikul et al. (2024). This study reflects the potential of φCIFT_MFB_MRSA12, φCIFT_MFB_MRSA28, and φCIFT_MFB_MRSA32 as effective antibacterial agents in phage therapy, given their stability across diverse environmental conditions and their promising host range capabilities.

The comparison of the three phage genomes against the NCBI genome database indicated that the top BLAST hits corresponded to the phages belonging to the genus *Silviavirus*. The broadest activity spectrum of the *Silviavirus* phages makes them highly desirable candidates for phage therapy applications (Sáez Moreno et al. 2021; Kolenda et al. 2022). These *Silviavirus* phages have been earlier reported from diverse sources including sewages, soil samples, and raw milk and exhibited a genome size ranging between 130 and 145 kb (Senevirathne et al. 2017; Lerdsittikul et al. 2024; Imklin et al. 2023). The genome sizes of the three phages used in this study—φCIFT_MFB_MRSA12, φCIFT_MFB_MRSA28, and φCIFT_MFB_MRSA32—ranged between 141,193 and 141,505 bp. The phages displayed a remarkable similarity in their genomes with an exception of few ORFs that differed among them. The variations in the host range exhibited by these phages can be attributed to these minor differences. Previous studies have also indicated a remarkable similarity (99–99.9%) between the genomes of the phages within the Herelleviridae family (Ajuebor et al. 2018; Kornienko et al. 2023).

Interestingly, there was a difference in the orientation of the ORFs among the three phages. In φCIFT_MFB_MRSA12 and φCIFT_MFB_MRSA32, 81.3% and 81.1% of the ORFs were on the negative strand, respectively, whereas for φCIFT_MFB_MRSA28, 80.6% of the ORFs were detected on the positive strand. The phages shared core genes characteristic of the Herelleviridae family including the genes encoding terminase large subunit, major capsid protein, portal protein, DNA primase, DNA polymerase I, helicases, RNA polymerase sigma factor, exonuclease, and resolvases. Each phage encoded RNA polymerase sigma factors and DNA polymerases. In the Herelleviridae family, phage-encoded DNA polymerase is essential for DNA replication, but the host RNA polymerase mediates transcription by binding phage-encoded sigma factors to initiate the predetermined cascade of phage gene expression (Barylski et al. 2020). Notably, no genes encoding tRNA were detected in the genome. The presence of bacterial virulence factors, or antimicrobial resistance-related functions or lysogenic cycle-related genes, was not detected in the phage genome thus minimizing concerns about the unintended spread of virulence or resistance factors and highlighting the therapeutic suitability of these phages.

The three phage genomes possessed five group I introns, the intragenic RNA sequences capable of self-splicing. Group I introns have been previously identified in Gram-positive bacteriophage genomes, indicating their occurrence to be a common feature within this group (Lavigne and Vandersteegen 2013; Senevirathne et al. 2017; Kornienko et al. 2023). The genomes of Herelleviridae family are known to vary in the size of DTRs, ranging from 1 to 16 kb (Barylski et al. 2020). All the three phage genomes harbored long direct terminal repeats (DTRs) of 10,893 bp at both ends, indicating a T5 phage-like packaging mechanism. This is consistent with the previous reports that DTRs of phages are similar between closely related phage species (Wang et al. 2005). ViPTree analysis of the three phages with the previously described phages from the Herelleviridae family, viz., *Lactobacillus* phage, *Bacillus* phage, *Listeria* phage, and *Enterococcus* phage, indicated that these phages share a common ancestry. Further, the three phages clustered separately from other Herelleviridae phages belonging to *Kayvirus*. All the phages were classified into the Bacillota host group reinforcing their taxonomic position. The combination of unique genomic features, such as long DTRs, varied ORF sequences, the presence of Group-I introns, and other RNA features like HEARO and RAGATH-18, in addition to their phylogenetic distinctiveness, underlines the significance of these phages as novel members of the Herelleviridae family.

A comparative analysis of the three phage genomes, alongside the phylogenetic analysis, was carried out to determine their taxonomic placement with respect to other species within the same family. According to the ICTV guidelines, members of the same species must exhibit a species threshold above 95%. The VIRDIC and taxMyPHAGE analysis predicted that the phages showed less than 95% similarity to other species within the *Silviavirus* genus strongly suggesting them to be a distinct species within the genus. To further support the classification, a Genome-BLAST Distance Phylogeny (GBDP) tree was generated using VICTOR. The analysis grouped the three phages as separate species within the *Silviavirus* genus. The three phages can be considered the same species as they exhibited a species threshold above 95% among them. In alignment with the ICTV guidelines for novel species identification, the phages differed by more than 5% from other closely related *Silviavirus* phages, consequently they have been assigned to a different species within the *Silviavirus* genus, highlighting their unique genetic and evolutionary identity.

Phage genomes are organized into distinct modules (locally collinear blocks), which can vary in configuration across other phages. To analyze these variations, a progressiveMauve analysis was used to align and compute locally collinear blocks (LCBs) between the three phages (φCIFT_MFB_MRSA12, φCIFT_MFB_MRSA28, and φCIFT_MFB_MRSA32) and the genomes of two other *Silviavirus* phages, namely, *Staphylococcus* phage KSAP7 and *Staphylococcus* phage vB_Sau-RP15, using default settings. The analysis revealed the presence of five LCBs in all the phages. The genome of φCIFT_MFB_MRSA28 was arranged in reverse complement orientation, similar to *Staphylococcus* phage KSAP7 and *Staphylococcus* phage vB_Sau-RP15, in contrast to φCIFT_MFB_MRSA32 and φCIFT_MFB_MRSA12 that were arranged in forward orientation.

To visualize the circular genome comparisons, BLAST Ring Image Generator (BRIG, version 0.95) was used. The genome of φCIFT_MFB_MRSA32, the longest among the three phage genomes, served as a reference to which all the other closely related *Silviavirus* phage genomes, φCIFT_MFB_MRSA12 and φCIFT_MFB_MRSA28, were compared. This comprehensive comparison provides insights into the structural and genomic similarities and variations among these closely related phage species.

The report highlights the identification and characterization of three novel *Silviavirus* phages with a high lytic potential from the South Asian geographical region. Notably, several countries including Japan, Belgium, China, Thailand, France, Taiwan, and South Korea have reported the discovery and study of *Silviavirus* phages. Owing to the specificity of phages, it is important to identify and characterize phages indigenous across particular geographical regions, given that the phages identified from a specific region may exhibit reduced effectiveness against bacterial strains from other parts of the world (Lerdsittikul et al. 2024). It is very important to establish a diverse and well-characterized collection of bacteriophages in the form of phage banks, which enables rapid screening and selection of the most suitable phages for therapeutic applications. This ensures effective and timely treatment against targeted bacterial strains (Lin et al. 2021; Nagel et al. 2022). The phages have been determined to have an optimum burst size of 75–107 PFU/C and are able to tolerate a range of temperatures (− 20–50 °C) and pHs (3–10) making them ideal candidates for phage-based applications. The genome study also indicates that the phages do not harbor any virulence-associated genes or lysogenic determinants. Comparative genome analysis found that the phages belonged to a novel group of *Silviavirus.* The phages could hold promise as biocontrol agents against MRSA strains prevalent in aquaculture systems to ensure seafood safety. The future success in phage-based applications will depend greatly on the development of strategies for the effective delivery of the phages and ensuring their long-term phage stability.

Reports have indicated that the replication of phages is limited in dried food matrices compared to moist foods due to lack of moisture that affects passive movement, which makes it necessary to optimize phage dosage with respect to different types of food matrices. Also, it is necessary to ensure adequate phage coverage to ensure their effectiveness (Moye et al. 2018; Shannon et al. 2020). The probable development of resistance could not be ignored; hence, mandatorily more than one phage should be used as consortia or cocktail of phages for the biocontrol purposes. Other limitations are their narrow stability in different pH and temperature, narrow period of shelf-life, and difficulty of stabilizing the phage formulations. The rapid escalation in phage research involving the application of genetic engineering techniques and effective delivery methods ensures more likelihood that the limitations are overcome thus ensuring their safety and efficacy in biocontrol applications. Regulatory approval in usage of bacteriophage for the biocontrol of food-borne pathogens in food production chain is very pertinent for wider application across the globe.

## Conclusion

In the present study, three lytic phages, viz., φCIFT_MFB_MRSA12, φCIFT_MFB_MRSA28, and φCIFT_MFB_MRSA32, were isolated and comprehensively characterized both phenotypically and genotypically for their safe and effective use in phage-based biocontrol strategies. The phages also represented novel species within the *Silviavirus* genus based on the ICTV species demarcation criteria threshold. The outcomes of the study indicate their remarkable stability across a wide range of pH levels and temperatures, which helps in their practical application across diverse environmental and clinical settings. The genome analysis revealed the presence of various phage-related genes with diverse functions including introns, RNAs with ribozyme like activities, HNH endonuclease-associated RNAs, and a repertoire of lytic enzymes. The comparative genome analysis indicated that the phages differed from each other with respect to a few ORFs like tail fiber proteins, Ig-domain proteins, and hypothetical proteins, which might contribute to the difference in the host range exhibited by these phages. This study is the first comprehensive report of the *Silviavirus* phages isolated from the South Asian geographical region, and helps in the advancement of phage-based therapeutics in the current situation of growing antimicrobial resistance. Further studies in animal models are required for the evaluation of the safety and effective therapeutic dose of the identified phages. This study provides a potential scope of the identified phages in the biocontrol of MRSA in seafood supply chains, which has profound implications in food safety and security.

## Data Availability

The genomic data generated in the project is submitted to public domain. Other primary data generated from this project is available with the corresponding authors and will be available based on the request.
